# *Schistosoma mansoni* infection and hepatitis B surface antigen carriage rate among school children in Jille Timuga District, Amhara Region, Northeast Ethiopia

**DOI:** 10.1371/journal.pntd.0012976

**Published:** 2025-04-08

**Authors:** Minwuyelet Maru Temesgen, Mengistu Legesse, Aklilu Feleke, Berhanu Erko, Hawa Worku, Birtukan Shiferaw, Anteneh Demelash, Nega Berhe

**Affiliations:** 1 Amhara Public Health Institute Dessie Branch, Amhara National Regional Health Bureau, Dessie, Ethiopia; 2 Aklilu Lemma Institute of Pathobiology, Addis Ababa University, Addis Ababa, Ethiopia; Central University of Tamil Nadu, INDIA

## Abstract

**Background:**

Hepatitis B virus (HBV) is highly prevalent and a major health problem in developing countries. Controversial findings are reported on the effect of schistosomiasis and HBV infection. This study aimed to describe the association of *S. mansoni* infection with Hepatitis B surface antigen (HBsAg) carriage rate in schistosome endemic setting.

**Methods:**

A cross-sectional study was conducted from January to March 2024 among school children aged 7–14 years old in two primary schools of Jille Timuga district of Oromo special zone, Amhara region, Ethiopia. Demographic and health related data was collected by Kobo collect tool. Blood and stool specimens were collected to test Hepatitis B infection using rapid test kit and *S.mansoni* infection by kato-katz method respectively. The data was analyzed by STATA version 17 statistical software. A descriptive statistic, bivariate and multivariate logistic regression analysis was used to identify associated factors. P-value of <0.05 was used as a cut-off in reporting statistical significance.

**Results:**

A total of 300 children participated in the study with a mean age of 10.5 years (±2) ranging from 7 to 14 years. Eighty-nine (29.6%) children were infected with *S. mansoni* and the sero-prevalence of hepatitis B surface antigen was 0.3%; no co-infection was observed. Children who had taken praziquantel mass treatment recently (<6 month) had higher infection rate at 34%. Likewise, highest prevalence of *S. mansoni* infection (39.8%) was found among 11–12 years age group. A significant association of sex with higher *S.mansoni* infection rate was observed where males had 2.07 increased odds of infection.

**Conclusions:**

The observed prevalence of *S. mansoni* infection (29.6%) was high in view of the ongoing preventive chemotherapy using praziquantel. The low, 0.3%, prevalence of HBV in the setting of higher *S.mansoni* prevalence underscore non well defined association of HBSAg carriage with schistosomiasis. However, a larger, well-controlled further research is recommended. The infection rate of *S. mansoni* was higher among children who recently took praziquantel which highlight the limitations of mass drug administration (MDA) program and possibility of re-infection. These emphasize the need for integrated schistosomiasis control programs, combining mass drug administration with other supportive intervention measures such as snail control.

## Author summary

We conducted a study in a rural district of Ethiopia to understand the relationship between a common parasitic infection, *Schistosoma mansoni*, and hepatitis B, a serious liver disease. We examined 300 schoolchildren to determine how many had *Schistosoma mansoni* and whether this infection increased their risk of carrying hepatitis B. Our results showed that nearly one in three children (29.6%) had *Schistosoma mansoni*, but only one child (0.3%) tested positive for hepatitis B. We found no cases where both infections occurred together, suggesting that *Schistosoma mansoni* does not necessarily increase the risk of hepatitis B in this population. We also observed that children who had recently received treatment for *Schistosoma mansoni* were still getting reinfected, highlighting the challenges of controlling the disease through medicine alone. This suggests that long-term solutions should include education, improved sanitation, and controlling the snails that spread the parasite. Our findings contribute to global health efforts by emphasizing the need for a more comprehensive approach to reducing parasitic infections in children. Future studies with larger groups of people in different areas will help confirm whether *Schistosoma mansoni* and hepatitis B are connected in other settings.

## Introduction

Hepatitis B virus (HBV) is a causative agent of both acute and chronic viral hepatitis. In HBV-endemic areas where at least 8% of the population is chronically infected, infections occur during the perinatal period or early in childhood followed by person-to-person contacts in household or schools [1]. Schistosomiasis is a parasitic infection that is second to malaria in prevalence and affects about 200 million people in over 70 countries, the great majority being in sub-Saharan Africa. According to the world health organization (WHO), about 251.4 million people required preventive treatment in 2021 [2]. Schistosomiasis is caused by *Schistosoma* species, of which *S. mansoni* is the most common. Often, the disease progresses to an advanced stage, called hepatosplenic schistosomiasis (HSS), which is frequently seen in endemic areas. In Ethiopia schistosomiasis is a significant public health concern with a national pooled prevalence of *S.mansoni* at 22% [3]. Over the past 15 years there has been a decline in *S. mansoni* prevalence, despite this progress, prevalence among school-age children is still high ranging between 24% [4], in Eastern Ethiopia, to 73.8% in southwest Ethiopia [5]. In Amhara region a significant variability of *S.mansoni* prevalence across different areas is observed ranging from 0.5% [6] to 60.7% [7].

Concurrent infection between hepatitis B virus and schistosomiasis is often observed in countries where schistosomiasis is endemic and might usually progress to chronic liver inflammation which might be due to host immune response. Schistosome infection has immunomodulatory effects that might influence outcome of hepatitis B infection. During intracellular infection, as of hepatitis B, Th1 immune response dominates, whereas during infection by extracellular pathogens such as *S. mansoni*, Th1 immune response is initiated during the early infection, and then it switches to Th2 dominant response that appears to be beneficial by limiting the spread of egg secretions and causing little or no parenchymal inflammation of the liver [8]. However, during concurrent infection, the Th2 dominant immune response towards *S. mansoni* might affect the clearance of hepatitis B virus and influence the extent and degree of the pathology and fibrosis or chronicity [9].

In addition, hepatitis B virus has a delayed amplification during replication unlike most viruses including HCV which enter a logarithmic phase of propagation immediately after infection [10]. In endemic areas, progresses of schistosoma infection to advanced stage HSS is likely due to failure of immunomodulation processes [11]. Studies reported increased susceptibility and progression of HBV in schistosomiasis co-infected subjects and the course of illness for those with Schistosoma-HBV infections are also found to be much more severe than for mono-infected subjects. Schistosome-HBV co-infection prolonged the carriage state and more often resulted in chronic hepatitis with greater cirrhosis, failure to treatment response as well as higher mortality which might be attributed to dominance of Th1immune response [12]. However, findings from mouse model reported quick clearance of acute HBV infection both in Th1-dominant acute phase and the chronic T regulatory dominant phase of schistosome infection which suggested the role of timing of infections [13,14].

Schistosome infections were also reported to impair immune responses including response of hepatitis vaccine [13]. In addition, studies that described the effects of praziquantel treatment on HBV disease course reported higher HBsAg carriage during persistent *S. mansoni* infection [15]. Although schistosomiasis and HBV co-infection pathogenesis remains controversial, in endemic area it might contribute to high HBV prevalence or severity of chronic liver inflammation. Higher carrier rate of HBsAg among patients with HSS is also reported than hepatointestinal schistosomiasis (HIS) which might be related to reduced viral clearance among co-infected cases [16]. Co-infection may also have an impact on disease pathophysiology as a marked depression in cell mediated immune responses was demonstrated during co-infection of HBV and/or hepatitis C virus (HCV) in a study from patients with hepatosplenic schistosomiasis [17]. Therefore, this study was aimed to describe the association of *S. mansoni* infection with HBsAg carriage rate and related factors in schistosome endemic setting.

## Materials and methods

### Ethics statement

Ethical approval was obtained from Aklilu Lemma Institute of Pathobiology Institutional Research Ethics Review Committee (ALIPB-IRERC) with reference number ALIPB IRERC/103/2015/23 and local ethical approval is obtained from Amhara Public Health Institute with reference number APHI/DB/4/1115/2015. Permission letter was obtained from local authorities. All study participants/mothers/guardians during the study period were informed the purpose of the study and their consent was sought formally from mothers/guardians in written for the study.

### Study area and period

The study was conducted from January to March 2024 among school children aged 7–14 years in two primary schools namely Gerbi Dire Gelma and Hadere elementary school. The study area is found in Jille Timuga district of Oromo special zone, Amhara regional state, Ethiopia which is located 270km from Addis Ababa, the capital city of Ethiopia and 170 km from Dessie.

### Study design

Cross-sectional study was conducted to screen the children for schistosomiasis using Kato-Katz method and HBV using rapid HBsAg kit. Kobo collect tool was used to assess socio-demographic characteristics, and identify possible risk factors related to transmission

### Study population and study participants

All school aged children living in the study area were the study population and selected children from the selected schools of Jille Timuga district were the study participants.

### Inclusion and exclusion criteria

Children aged 7–14 years of age were included, while those with chronic or other diagnosed diseases, as well as those outside this age range, were excluded.

### Sample size determination and sampling method

Sample size was calculated based on single population sample size estimation formula [18] using Epi Info v 7.2.5.0 statistical tool with the following assumptions; 99% conﬁdence level, 80% power, 0.5 ratio of unexposed to exposed, 9.6% of HBV/schistosome co-infection prevalence [16] and 3.8 odds ratio [19]. After adding a 20% nonresponse rate we obtained a minimum sample size of 300. A list of all eligible children aged 7–14 years was taken from the two selected schools for sampling frame, and proportion-to-size allocation was made to determine the required sample size from each school. Then, a systematic random sampling technique was applied to select the required numbers of children from each school.

### Data collection

Two nurses and two laboratory technicians from nearby health centers (Senbete and Gerbi) and one supervisor from Amhara Public Health Institute, Dessie Branch (APHI DB) were recruited and trained for the data collection. Scio-demographic and health related factors were collected by the nurses using Kobo collect data collection tool through face to face interviewing each one of the children and mothers/guardians. Then, blood sample was collected from finger prick and immediately tested for HBsAg. The middle or ring finger was cleaned and pricked, then after wiping off the first drop, free floating drop was used to the test. The laboratory technicians collect stool specimen using clean, waterproof cup for Kato-Katz technique.

### Laboratory methods

For parasitological examination, two smears were prepared from each specimen and examined by Kato-Katz technique. The feces are pressed through a mesh screen to remove large particles and a portion of the sieved sample is then transferred to the hole of a template on a slide. After filling the hole, the template is removed and the remaining sample is covered with a piece of cellophane soaked in malachite green containing glycerol. The glycerol ‘clears’ the fecal material around the eggs. The eggs are then counted and the number calculated per gram of faces.

To test for hepatitis B surface antigen, blood sample was tested using SD Bioline HBsAg rapid test kit. The free-floating drop was applied to the test pouch by gently massaging the finger. Then, two drop of buffer was added and the result was read according to the manufacturer’s instruction.

### Quality assurance

The questionnaire used for the study was pretested in 5% of children in a school located in Kombolcha, a nearby town and necessary corrections was made. A one-day training was given for data collectors and strict supervision was done to check adherence of the procedures for both stool examination and blood test. All the Kato-Katz smears were sent to Amhara Public Health Institute Dessie Branch and rechecked by parasitologists.

### Data analysis

Data was exported into excel and imported into STATA version 17 statistical software for statistical analysis. A descriptive statistic, bivariate and multivariate logistic regression models were used to summarize the data, and to investigate associations. P-value of <0.05 was used as a cut-off in reporting statistical significance. Outlier detection was performed using box plot and interquartile range (IQR) method. No extreme values were identified in the dataset, confirming that all observations fell within the expected range. Wald’s test was used to assess the logistic regression model, and the significance of individual predictors were evaluated by examining their p-values where sex showed more strong prediction, X^2^ = 3.29 (p=0.06) followed by age, contact with river and time of praziquantel treatment. The Likelihood Ratio (LR) test was also used to compare the fit of the logistic models with a p<0.05 indicating a significant improvement in model fit and the model which fits best was used. For evaluating discriminatory power, we calculated sensitivity (true positive rate) and specificity (true negative rate) from the confusion matrix and in overall 70.3 were correctly classified, while the Area Under the Receiver Operating Characteristic Curve (AUC-ROC) score which provides a summary measure of the model’s ability to distinguish between classes, was >0.6 which is above average as value closer to 1 indicating better performance. Multicollinearity among regressors was also assessed using the Variance Inflation Factor (VIF) and all values were below the accepted threshold of 5 with a mean VIF of 1.05 which shows low multicollinearity and it is not a significant concern.

## Results

### Socio-demographic data

A total of 300 children participated in the study with 100% response rate. As indicated in Table 1, there are more males, 174 (58%) than females, 126 (42%) among the children with a mean age of 10.5 years (±2) ranging from 7 to 14 years (Table 2).

**Table 1:** Socio-demographic characteristics of children and mothers, Jille Timuga district, Amhara Region, Ethiopia, 2024.

Variables	Frequency	Percentage
**Children**		
Sex		
Males	174	58
Females	124	42
Age group		
<10 years	103	34.3
11-12 years	123	41
>12 years	74	24.7

**Table 2:** Prevalence of Schistosoma infection, Jille Timuga district, Amhara Region, Ethiopia, 2024.

Variable	*S. mansoni*		Pearson chi2 (p)
**Yes, n (%)**	**No, n (%)**	
Sex			
Males	53 (30.5%)	121 (69.5)	0.12 (0.74)
Females	36 (28.6%)	90 (71.4%)	
Age group			
<10 years	23 (22.3%)	80 (77.7%)	10.34 (0.006)
11-12 years	49 (39.8%)	74 (60.2%)	
>12 years	17 (22.9%)	57 (77.1%)	
Contact with river during the previous week of data collection			
Yes	46 (30.7%)	104 (69.3%)	0.14 (0.70)
No	43 (28.7%)	107 (71.3%)	
Time of Praziquantel treatment			
< 6 months	34 (34%)	66 (66%)	2.21 (0.33)
6 to 12 months	14 (22.9%)	47 (77.1%)	
> 1 year	7 (29.1%)	17 (70.8%)	
Total	89 (29.7%)	211 (70.3%)	

### *S* *. mansoni* infection

All 300 children included in the study provided stool specimen and 89 (29.6%) were infected with *S. mansoni*. As shown in Table 2, slightly higher proportion of males, 30.5%, than females, 28.6%, were infected, whereas, there was a comparable proportion of infected children who had current contact with river and who had not contact in a week before sample collection. Children who had taken praziquantel mass treatment recently (< 6 month) had higher infection rate at 34%. Likewise, highest prevalence of *S. mansoni* (39.8%) was found among 11–12 years age group. The Chi-square test showed a significant association between age group and *S. mansoni* prevalence, with higher infection rates in the 11–12 years age group. However, no significant association was found between sex and infection or between recent river contact and infection. Other helminth infection, such as hookworms*, E. vermicularis*, and *Taenia* species were also identified at a prevalence of 1.1% each.

### Hepatitis B virus infection

The sero-prevalence of hepatitis B virus was 0.3%, only one child was positive for HBsAg among the tested 300 tested. However, 11 (3.7%) reported previous hepatitis diagnosis. The infected child was a 12 years male, and not co-infected with schistosomiasis. Regarding health-related factors associated with hepatitis B infection, the majority, 41% of children were born second and about 40% had 4–5 siblings. Traditional practices were reported from 86 (28.7%) children. (Table 3).

**Table 3:** Health related factors associated with hepatitis B infection among children, Jille Timuga district, Amhara Region, Ethiopia, 2024.

Variables	Frequency	Percentage
Birth order		
1^st^	72	24
2^nd^	123	41
3^rd^	58	19.3
4^th^	22	7.3
5^th^	25	8.4
Number of siblings		
<=3 siblings	65	21.7
4 to 5 siblings	120	40
6 to 7 siblings	78	26
>=8 siblings	37	12.3
History of traditional practice		
Yes	86	28.7
No	214	71.3
Previous hepatitis diagnosis		
Yes	11	3.7
No	289	96.3

### Factors associated with *S.mansoni* infection

Associations between risk factors and *S. mansoni* infection from logistic regression models are shown in Table 4. A significant association of sex with higher *S. mansoni* infection rate was observed where males had increased odds of infection when compared to females (AOR = 2.07; 95% CI: 1.03 - 4.16). Higher infection rates of *S. mansoni* were more likely to be observed among age groups between 11–12 years (AOR = 2.05; 95% CI: 0.98 - 4.27) and among children who had a recent contact with river for bathing or washing clothes. Participants, who have been treated with praziquantel recently (< 6 months) tended to have higher odds of infection with *S. mansoni* (AOR=1.15; 95% CI: 0.40 - 3.31).

**Table 4:** Bivariate and multivariate logistic regression analysis of *S. mansoni* infection with sociodemographic and health related factors among children, Jille Timuga district, Amhara Region, Ethiopia, 2024.

Variables	COR (95% CI)	P-value	AOR (95% CI)	P-value
Sex				
Males	1.09 (0.66 - 1.81)	0.724	2.07(1.03 - 4.16)	*P=0.04*
Females	Ref			
Age group				
<10 years	Ref			
11-12 years	2.03 (1.27 - 4.14)	0.005	2.05 (0.98 - 4.27)	*P=0.054*
>12 years	1.03 (0.50 - 2.11)			
Contact with river (recent contact)				
Yes	1.10 (0.67 - 1.80)	0.705	1.33 (0.51 - 3.5)	*P=0.5*
No	Ref			
Time of Praziquantel treatment				
< 6 months	1.25 (0.47 - 3.30)	0.652	1.15 (0.40 - 3.31)	*P=0.78*
6 to 12 months	0.72 (0.24 - 2.09)			
> 1 year	Ref			

## Discussion

The relation between schistosomiasis and HBV carriage is not well defined and varied among different findings. In some studies, schistosome infection has been linked to increased susceptibility, carriage rate and exacerbated liver damage [16,20]. Other studies challenge the existence of a direct association [21]. In the present study the prevalence of HBV infection was low, only 0.3%, and no co-infection was observed. This finding contrasts with a study in Sudan that reported a significantly higher HBV carriage rates, about 25%, among schistosomiasis compared to those without schistosomiasis [22]. Similarly, research in Egypt [9] and China [23] revealed that co-infected individuals were more likely to remain HBV carriers. On the other hand, Studies from Sudan and Nigeria have reported a lack of clear association between *S. mansoni* and HBV infections [24]. However, in our study the lower prevalence of HBV could partly be due to lower exposure of HBV in the area, which in turn implies decreased necessity of one to the other for co-existence.

The prevalence of *S. mansoni* infection among the children in this study was 29.6%, which reflects a significant public health burden in the community. The findings are much higher than the global 14.8% prevalence of endemic regions, and 15.3% of sub-Saharan Africa [25]. A study in Ethiopia also reported a pooled prevalence of 28.7% among school-aged children ranged from 14.9% in Afar region to 39.7% in Southern region [26]. Similarly, other African countries such as Uganda reported a prevalence of 36% [27]. This level of prevalence suggests ongoing transmission and high environmental contamination with *S. mansoni* eggs.

Studies have shown that schistosomiasis is more prevalent in regions with lower educational attainment and socioeconomic challenges [28]. These factors likely contribute to increased exposure to schistosome infections. Similar findings have been reported in African countries, where knowledge was associated with higher exposure [29] and also in Ethiopia where it was associated with undernutrition [30].

The present study observed a significant association of gender for schistosome infection, where males had 2.07 times more odds of infection than females. This could reflect cultural preferences for male children to play out doors and contact with infected water. Similar gender discrepancies have been also reported from a review that systematically investigate and quantify the differences in Schistosoma infection burdens between males and females in Africa that revealed 1.15 (95% CI 1.08–1.22) higher odds in males [31].

In our study children who had taken praziquantel mass treatment recently (< 6 month) had 1.15 times higher infection rate. This suggests several important considerations about the dynamics of reinfection and the effectiveness of mass drug administration (MDA) programs in schistosomiasis control. One plausible explanation for this trend is the rapid reinfection that occurs in endemic areas. Interestingly, the higher infection rate among those treated recently may also reflect exposure to residual infections, that were not completely cleared by treatment, due to factors such as suboptimal dosing, treatment adherence issues, or drug-resistant schistosome strains in the population. This notion is also supported in our finding by the higher proportion of infected children who had recent water contacts than their counterparts, 30.7% vs 28.7% respectively. A study in Uganda also found children in high-transmission settings exhibited rapid reinfection within months of praziquantel treatment, with infection rates returning to pre-treatment levels within a year [32].

## Limitations of the study

The study does not analyze immune markers or immune status, which could provide insights into host susceptibility and response to infections.

## Conclusions and recommendations

The prevalence of *S. mansoni* in this study was 29.6%, which is unacceptably high. Our findings also indicate that the infection rate was higher among children who had recently taken praziquantel, highlighting the limitations of the mass drug administration program. Similarly, the higher prevalence among children who took the drug a year ago suggests a high risk of re-infection. These findings emphasize the need for integrated schistosomiasis control strategies that go beyond MDA by incorporating sustained health education, improved water, sanitation, and hygiene infrastructure, and behavioral interventions in schools.

In rural settings like ours, where water contact is frequent, strengthening school-based health programs, improving maternal literacy, and engaging community leaders in disease prevention efforts could significantly reduce transmission. Additionally, mapping geographic hotspots within the community can help target interventions more effectively, maximizing the impact of limited resources. Environmental measures, such as snail control programs and the promotion of safe water sources, should also be prioritized.

The low prevalence of HBV (0.3%) in our study is consistent with rates in the general population and therefore, this infection rate in the setting of higher *S.mansoni* prevalence underscore the absence of association of HBV with schistosomiasis. However, larger, well-controlled further research is recommended by including areas with high HBV prevalence to explore this relationship more comprehensively.
